# Successful resection of intrahepatic cholangiocarcinoma with idiopathic thrombocytopenic purpura using thrombopoietin receptor agonist: a case report

**DOI:** 10.1186/s40792-019-0619-4

**Published:** 2019-04-10

**Authors:** Kouki Hoshino, Norifumi Harimoto, Ryo Muranushi, Kenichiro Araki, Takahiro Yamanaka, Kei Hagiwara, Norihiro Ishii, Mariko Tsukagoshi, Takamichi Igarashi, Hiroshi Tanaka, Akira Watanabe, Norio Kubo, Takehiko Yokobori, Ken Shirabe

**Affiliations:** Department of Hepatobiliary and Pancreatic Surgery, Graduate School of Medicine, Gunma University, 3-39-22, Showa-machi, Maebashi, Gunma 371-8511 Japan

**Keywords:** Idiopathic thrombocytopenic purpura, Intrahepatic cholangiocarcinoma, Thrombopoietin receptor agonist, Left hemi-hepatectomy

## Abstract

**Background:**

Patients with idiopathic thrombocytopenic purpura (ITP) have low platelet counts and an increased risk of complications. Therefore, these patients generally require high-dose immunoglobulin therapy and platelet transfusion. However, thrombopoietin receptor agonists (TPO-RAs) have recently become available for use in the preoperative treatment strategy for intractable ITP. Recent studies have also reported radiofrequency ablation (RFA) or tissue biopsy as perioperative management for thrombocytopenia using TPO-RA. However, no report has described the use of TPO-RA in a case of hepatectomy.

**Case presentation:**

A 76-year-old man presented with intrahepatic cholangiocarcinoma (IHCC) complicated with ITP. His platelet count was 3.5 × 10^4^/μL. To increase platelet levels prior to surgery, romiplostim was administered subcutaneously (70 μg per week for 3 weeks) and eltrombopag was administered orally (25 mg per day for 23 days), as TPO-RA. His platelet count increased to 14.1 × 10^4^/μL. The patient was successfully and safely treated with left hemi-hepatectomy and TPO-RA as preoperative platelet management.

**Conclusions:**

This case suggests that TPO-RA can be effective, and could serve as a new treatment option in the preoperative management of ITP.

## Background

Idiopathic thrombocytopenic purpura (ITP) is a rare autoimmune disorder in which the patient’s immune system reacts with a platelet autoantigen. As a result, thrombocytopenia occurs due to immune-mediated platelet destruction and/or suppression of platelet production [1, 2].

The main treatment methods for ITP include administration of *Helicobacter pylori*-eradicating agent, prednisolone, immunoglobulin, and immunosuppressive agent, or splenectomy. Specifically, for ITP patients requiring more invasive procedures, such as surgery, platelet transfusion or immunoglobulin therapy is performed before invasive procedures.

However, in recent years, thrombopoietin receptor agonists (TPO-RAs) that stimulate platelet production, including romiplostim and eltrombopag, have been adopted as a treatment strategy. TPO is produced in the liver and kidney and is a growth factor that promotes increase in megakaryocytic differentiation. Moreover, TPO-RA can increase platelet counts by promoting megakaryocyte hematopoiesis.

Herein, we present the case of a 76-year-old man with intrahepatic cholangiocarcinoma (IHCC) complicated with ITP. The patient was successfully and safely treated with left hemi-hepatectomy and TPO-RA as preoperative platelet management.

## Case presentation

A 76-year-old man was referred to our hospital with IHCC. Five years earlier, he had been treated for ITP at another hospital, and had been administered prednisolone 5 mg/day. At the time of ITP diagnosis in the prior hospital, the value of the platelet-associated IgG (PAIgG) was elevated and all of the anti-phospholipid antibody syndrome (APS)-related antibodies were negative. In addition, only the number of the megakaryocytes increased in bone marrow aspiration, which suggests the hematopoietic efficacy in bone marrow was maintained. *Helicobacter pylori* was previously eradicated in the other hospital.

His past medical history was otherwise unremarkable. More recently, his sister had been diagnosed with pancreatic cancer, and he therefore requested a tumor marker evaluation. The evaluation revealed elevated carbohydrate antigen 19-9 (CA19-9), and a liver tumor was detected by computed tomography (CT). Thus, he was referred to our hospital for detailed examination and treatment for the liver tumor.

**Fig. 1:**
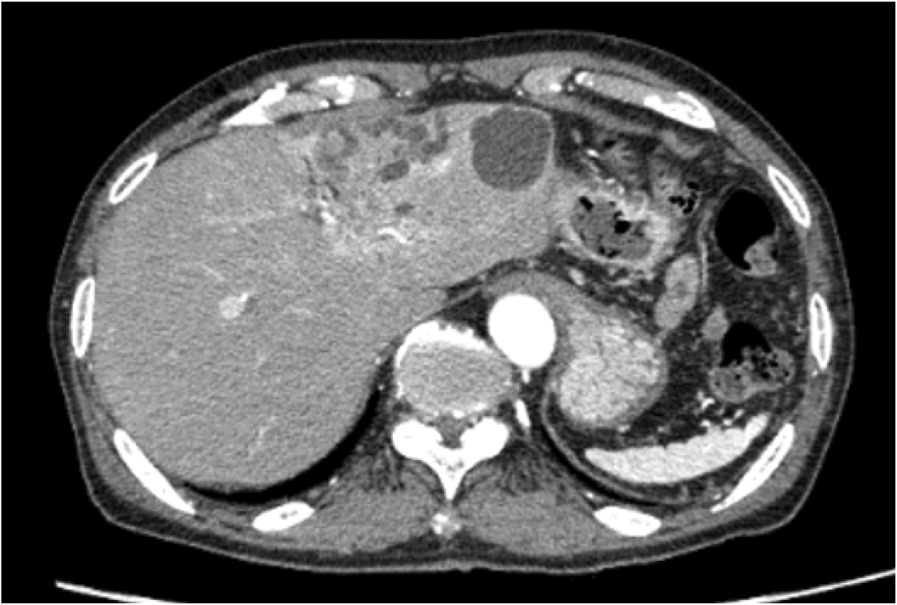
Computed tomography images showing a tumor in segment 3 (yellow arrow). The peripheral bile duct branch of segment 3 was dilated

Multidetector row CT revealed a liver tumor that was 25 mm in diameter and had a low density with poor enhancement. The peripheral bile duct branch of segment 3 was dilated; therefore, we suspected the periductal infiltrating type of IHCC (Fig. 1), which prompted us to perform left hemi-hepatectomy.

**Fig. 2:**
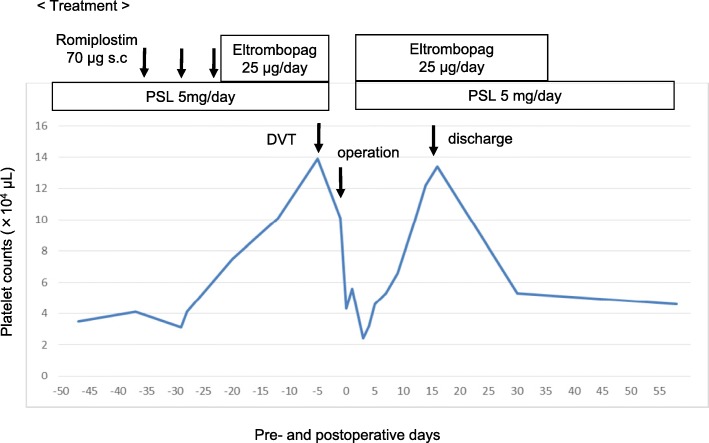
Clinical course with platelet counts and treatment using thrombopoietin receptor agonist (TPO-RA)

His laboratory findings on admission are summarized in Table 1 and included a platelet count of 3.5 × 10^4^/μL. To increase platelet levels prior to surgery, romiplostim was administered subcutaneously (70 μg per week for 3 weeks) and eltrombopag was administer orally (25 mg per day for 23 days), as TPO-RA. Because oral medications we are more convenient, romiplostim was switched to eltrombopag. His platelet count increased to 14.1 × 10^4^/μL (Fig. 2). After starting eltrombopag, fibrin degradation product (FDP) and d-dimer levels were slightly elevated. Therefore, we performed lower limb venous ultrasonography, which revealed venous thrombosis in the veins of both soleus muscles. We consulted with cardiovascular medicine and hematology teams about our patient’s case. Because his thrombosis was venous rather than arterial, it was thought to be largely unrelated to the platelet increase. Accordingly, the patient’s cancer was suspected to have caused his thrombosis, via increased activation of clotting factors. In addition, we speculated that the risk of the thrombus in the soleus muscles causing pulmonary thromboembolism was extremely low. Thus, eltrombopag was discontinued. After eltrombopag cessation, the platelet count slightly decreased to 10.0 × 10^4^/μL, but remained at a sufficient level; subsequently, the operation was performed on schedule (Fig. 2).

**Table 1:** Laboratory data on admission

	Value
WBC count, /μL	4200
RBC count, × 10^4^/μL	401
Hb, g/dL	13.2
Ht, %	38.7
PLT, ×10^4^/μL	3.5
PT, %	123
PT-INR	0.89
APTT, sec	25.2
T-bil, mg/dL	0.6
AST(GOT), IU/L	23
ALT(GPT), IU/L	17
ALP, IU/L	147
γ-GTP, IU/L	38
Amy, IU/L	56
TP, g/dL	6.4
Alb, g/dL	4.2
BUN, mg/dL	17
Cre, mg/dL	1.10
Na, mEq/L	140
K, mEq/L	4.2
Cl, mEq/L	103
Ca, mg/dL	9.1
CRP, mg/dL	0.02
HbA1c, %	5.7
CEA, ng/mL	1.5
CA19-9, U/mL	130
AFP, ng/mL	1.8
HBs-Ag	(−)
HBs-Ab	(−)
HBc-Ab	(−)
HCV-Ab	(−)

*AFP* α-fetoprotein, *ALB* albumin, *ALP* alkaline phosphatase, *ALT* alanine aminotransferase, *APTT* activated partial thromboplastin time, *AST* aspartate aminotransferase, *BUN* blood urea nitrogen, *CA19-9* carbohydrate antigen 19-9, *CEA* carcinoembryonic antigen, *Cre* creatinine, *γ-GTP* c-glutamyl transpeptidase, *Hb* hemoglobin, *Hct* hematocrit, *LDH* lactate dehydrogenase, *Plt* platelets, *PT* prothrombin time, *RBC* red blood cell, *T*-*bil* total bilirubin, *TP* total protein, *WBC* white blood cell

We performed left hemi-hepatectomy combined with left-sided caudate lobectomy and regional lymphadenectomy, because the lymph nodes around the common hepatic artery were larger than the preoperatively measured size. The operative time was 412 min, and blood loss was 395 mL, requiring no transfusion. Pathological findings were cholangiocellular carcinoma of the mass forming type, and bile duct infiltration type.

Although his platelet count decreased to 4.3 × 10^4^/μL just after the operation, eltrombopag and prednisolone were administered on the following day. His platelet count decreased to 2.4 × 10^4^/μL at postoperative day (POD) 3, but increased gradually thereafter, which made blood transfusion unnecessary. Drainage characteristics were not remarkable and bleeding and bile leakage was not observed after the operation. Although chylorrhea developed on POD 8, it was improved through the use of a fat-restricted diet. The drain was removed on POD 14, and he was discharged from the hospital on POD 18 without complications (such as bleeding, sepsis, pneumonia, or portal vein thrombosis). Eltrombopag was continued until POD 37. The platelet count decreased to 3–4 × 10^4^/μL again, which was the same as the preoperative level. However, rebound phenomenon requiring platelet transfusion was not observed.

## Discussion

ITP is characterized by thrombocytopenia and purpura resulting from the binding of platelets by autoantibodies directed toward platelet surface glycoproteins (GPs), especially GP IIb/IIIa and the GP Ib/IX complex, leading to their clearance by the reticulo-endothelial system [1–3]. However, it has recently been reported that ITP is not necessarily accompanied by the purple spots that are believed to indicate ITP. In some other countries, ITP is called primary immune thrombocytopenia. There are some reports of solid carcinoma cases complicated by ITP [4–7], but they show involvement of the gynecological or alimentary systems, such as the esophagus, stomach, and colon. Krauth et al. reported that there were no cancer sites found in the liver in ITP-associated cancer patients [7]. Further, using the search terms “idiopathic thrombocytopenic purpura” and “intrahepatic cholangiocarcinoma” in PubMed resulted in no case reports of intrahepatic cholangiocarcinoma with ITP.

ITP is characterized by common bleeding disorders. Previously, it was reported that ITP develops mainly in women aged 20–40 years; however, recent epidemiological studies revealed that, besides in younger women, ITP also has a tendency to develop in middle-aged or older men and women [8–10]. These recent data would be expected to increase the reported cases of ITP among patients with solid carcinoma. Moreover, therapeutic drugs for ITP, such as steroids or immunosuppressive drugs, could influence carcinogenesis or cancer treatment.

In Japan, when *Helicobacter pylori* infection is ruled out or eradication therapy is invalid in an ITP patient, steroid therapy is generally chosen as the first-line therapy. Conversely, splenectomy is often performed as the second-line therapy when a patient is at risk for serious side effects from steroid use, or steroid therapy is not deemed an appropriate therapy. It has previously been reported that platelets increase in 60–70% of those who have undergone splenectomy [11]. Recently, laparoscopic splenectomy, which is more minimally invasive than open surgery and may help to reduce the bleeding, increase than before by the progress in the laparoscopic surgical techniques.

When invasive surgeries are considered for ITP patients, low platelet counts present a critical problem. These patients are at an increased risk of life-threatening complications, such as massive hemorrhage. Thus, perioperative management for low platelet count in ITP patients is very important. In addition, most ITP patients have been on long-term steroid therapy, which could cause glucose tolerance, peptic ulcer disease, delayed wound healing, suture leakage, abscess in the abdominal cavity, acute renal failure, sepsis, and pneumonia. According to the consensus-based recommendation for target platelet counts during surgery in adults, the preoperative platelet count should be ≥ 5.0 × 10^4^/μL for minor surgeries, ≥ 8.0 × 10^4^/μL for major surgery, and ≥ 10.0 × 10^4^/μL for major neurosurgery [12]. However, hepatectomy is associated with an increased risk of bleeding and, in the presented case, the target platelet count was set at 10.0 × 10^4^/μL to ensure hemostasis.

To increase the number of platelets before an invasive surgery, high-dose intravenous immunoglobulin (IVIG) therapy is commonly used. To date, there have been few reported ITP cases in which high-dose IVIG therapy was administered before an invasive surgery [13–15]. Imbach et al. [16] suggested administering 400 mg/kg/day of complete molecular model immunoglobulin for 5 days. After the therapy is started, platelet levels begin to increase at the third or fourth day and reach a high level in approximately 1 week. The effect is transient but is considered to continue for 2–4 weeks. Adverse effects include chills, rigor, fever, headache, and rash. Rare but serious complications include shock, anaphylactoid symptom, liver function disorder (with an incidence rate of less than 0.1–5%), aseptic meningitis, acute renal failure, thromboembolism, pancytopenia, and lung edema [17–19]. Although the above mentioned adverse effects are approximately equally as likely to be detected, high-dose IVIG therapy can increase the platelet count relatively safely. However, this treatment is very cost intensive (Table 2). When platelets do not adequately increase by this method (effectiveness rate 70–90%) [20, 21], platelet transfusion may be required. In addition, the risk of contracting infectious diseases, such as human immunodeficiency virus, hepatitis C virus, hepatitis B virus, and human T cell lymphotrophic virus type 1 infections, is a substantial clinical problem [12]. As a means of avoiding high costs, risks of infectious diseases, and platelet blood transfusion, TPO-RA can be used to restore the perioperative platelet level. Recently, TPO-RA was authorized in Japan as a therapeutic drug for refractory ITP, and romiplostim and eltrombopag are widely available. Regarding the method of administration, romiplostim is administered as a weekly subcutaneous injection, and eltrombopag is administered daily as an oral preparation. In terms of increasing platelet levels, the actions of these drugs are dose-dependent. The number of platelets begins to increase 5–7 days after the initiation of treatment, and the maximum platelet increase is observed in 12–16 days. Moreover, continued use can steadily increase the platelet count. TPO-RA is effective for more than 80% of intractable cases [4], and the platelet count increases by 5.0 × 10^4^/μL, decreasing the risk of bleeding. Table 2 compares the characteristics of high-dose IVIG therapy and TPO-RA. Both the time for the platelet increase to begin as well as to reach the maximum is longer in TPO-RA (12–16 days) than in IVIG therapy (7 days) (Table 2). As such, we must be aware of this increase in time, and if there is an emergency such as bleeding, abdominal pain, or fever, we should defer to established conventional methods. In these cases, IVIG therapy or platelet transfusion would be more appropriate treatment modalities.

**Table 2:** Comparison of high-dose intravenous immunoglobulin (IVIG) therapy and thrombopoietin receptor agonist (TPO-RA)

Characteristic	IVIG therapy	TPO-RA
Time of the beginning of the increase	3–4 days	5–7 days
Time before reaching the maximum	7 days	12–16 days
Effective rate	70–90%	80%
Validity	2–4 weeks	2 weeks
Rate of platelet transfusion avoidance	(−)	72%
Adverse events	Headache, fever, nausea	Cytopenia
	Muscle pain, general malaise	Arterial and venous thrombosis
	Aseptic meningitis	Bone marrow reticulin fibrosis
	Acute renal failure	Rebound thrombocytopenia after discontinuation (platelet decrease relative to baseline)
	Infection (HCV, HIV, etc.)	Increases of ALT concentration
	Thrombosis	Headache
	Cerebral infarction	
	Pulmonary infarction	
Cost	Venoglobulin IH® 5% IV	REVOLADE® Tablets 12.5 mg 22.61 USD/tablet
	5 g/100 mL = 349.62 USD/vial	REVOLADE® Tablets 25 mg 44.55 USD/tablet
	In case, 400 mg/kg/day, 50 kg	In our case, preoperative for 23 days, 25 mg/day
	349.62 × 4 vial × 5 days	+ postoperative 30 days
	= 6992.35 USD	= 2396.96 USD

*ALT* alanine aminotransferase, *IV* intravenous, *HCV* hepatitis C virus, *HIV* human immunodeficiency virus, *USD* United States dollars

The present case was not an emergency case, and thus, we selected TPO-RA. However, fatal complications, such as liver function disorders, thrombosis, osteomyelofibrosis, and rebound phenomenon (a further drop from the baseline platelet count), have been reported [22–24]. In particular, thrombosis is a critical complication. In a phase 3 clinical study evaluating the long-term safety and efficacy of eltrombopag in adults with ITP [24], the major adverse effects reported with the TPO-RA were liver function disorder (15%; 45/302), a thromboembolic event (6%; 19/302), included such as deep vein thrombosis, pulmonary embolism, and myocardial or cerebral infarctions. In addition, a previous study [22] of cirrhosis and thrombocytopenia reported that eltrombopag increased the risk of portal vein thrombosis (4%; 6/143). While this was not a direct study of ITP, it does provide additional evidence that eltrombopag can cause serious side effects in some patients. Particularly in liver surgery, liver function disorder (15%) [24] and portal vein thrombosis (4%) [22] are adverse effects of TPO-RA and may have a serious influence on preoperative liver function. These factors carry sufficient risk for some patients that surgery may be postponed, or even deemed impossible. Additionally, it is necessary to be extra vigilant in monitoring for the risk of postoperative portal vein thrombosis. In the present case, deep vein thrombosis developed in the veins of both soleus muscles. After our discussion with the cardiovascular medicine team, it was decided that the platelet increase was unlikely to be related to the thrombosis, since it was venous rather than arterial. Accordingly, we hypothesized the patient’s cancer was the primary cause, but it cannot be ruled out that it was possibly a side effect of TPO-RA. Thrombosis is regarded as being attributable to platelet increases and platelet activation, but it is not certain to develop, regardless of the platelet count [12, 22]. Even for our case, we were able to detect the venous thrombosis in the lower limb vein in both soleus muscles early because of the slight elevation of FDP and D-dimer levels. Therefore, we should be careful during blood sample collection and regularly perform ultrasonography to detect thrombus in a timely manner. In addition, a point of concern in our case report is that platelet count dropped to 2.4 × 10^4^/μL at POD3, despite the TPO-RA and steroid treatments being started at POD1. We considered a platelet transfusion at this point, but performed a thorough follow-up and found no progression of anemia or any symptoms of bleeding from the drains. The cause of the drop in platelet count after the operation therefore remains unclear. However, one reason that may have influenced platelet count was the invasive surgical technique. We also hypothesize that the platelet count increases seen with TPO-RA may have a short, transient, or weak response. Regardless of the direct cause, if the symptoms are present, clinicians should not hesitate to perform a platelet transfusion.

Recently, TPO-RA has been used for the management of chemotherapy-induced thrombocytopenia [25] and before radiofrequency ablation (RFA) [26]. However, the long-term effect of TPO-RA on malignant tumors is still unknown, and thus, it should be used with caution. Moreover, even operations for abdominal malignant tumors can increase the risks of deep vein thrombosis and pulmonary vein embolism, and these risks could be magnified by the use of TPO-RA during the preoperative period, because it is expected to increase thrombotic tendencies. Therefore, physicians should demonstrate special caution regarding the adverse effects of deep vein thrombosis and pulmonary vein embolism during the perioperative period. It has been suggested that there is a high risk of inducing thrombosis in a patient with certain risk factors, such as anti-phospholipid antibody syndrome or prior cases of thrombosis, and as such clinicians need to carefully determine if the application of TPO-RA is advisable in individual patients.

## Conclusion

We presented a case of IHCC complicated with ITP, which was successfully and safely treated with left hemi-hepatectomy and TPO-RA as preoperative platelet management. Altogether, the findings of this case suggest the effectiveness of TPO-RA, which could be a new treatment option in the preoperative management of ITP.

## Abbreviations

CA19-9: Carbohydrate antigen 19-9
CT: Computed tomography
FDP: Fibrin degradation product
GP: Glycoprotein
IHCC: Intrahepatic cholangiocarcinoma
ITP: Idiopathic thrombocytopenic purpura
IVIG: Intravenous immunoglobulin
POD: Postoperative day
RFA: Radiofrequency ablation
TPO-RA: Thrombopoietin receptor agonist
